# Ciliated foregut cyst in the triangle of Calot: the first report

**DOI:** 10.1186/s40792-016-0147-4

**Published:** 2016-02-25

**Authors:** Osama S. Al Beteddini, Nasir K. Amra, Emad Sherkawi

**Affiliations:** Surgery Department, Johns Hopkins Aramco Healthcare/Dhahran Health Centre, Saudi Aramco, PO Box 76, Dhahran, 31311 Saudi Arabia; Pathology Department, Johns Hopkins Aramco Healthcare/Dhahran Health Centre, Saudi Aramco, PO Box 76, Dhahran, 31311 Saudi Arabia

**Keywords:** Ciliated, Foregut, Cyst, Gallbladder, Cholecystectomy

## Abstract

Ciliated foregut cysts are rare anomalies arising from remnants of aberrant embryological development. Around 100 reports on the presence of these congenital masses in the tracheobronchial tree, mediastinum, liver, pancreas and, rarely, the gallbladder have been described. In this article, the case of a 33-year-old woman, who was operated for a laparoscopic cholecystectomy, is presented. During the dissection of the triangle of Calot, a cystic mass, attached to the common hepatic duct, was discovered incidentally. This cyst was dissected off the hepatic duct, and no communication between both structures was found. The histopathological diagnosis was consistent with a ciliated foregut cyst. The postoperative course was uneventful. After reviewing the literature on this pathological entity, we found that this is the first report of a ciliated foregut cyst that is located in the triangle of Calot and found separate from the biliary structures, the gallbladder and the liver. We present a review of the literature on this entity, discussing diagnostic measures and therapeutic options.

## Background

The presence of cysts originating from the embryonic primitive foregut and containing a respiratory-type epithelial lining has rarely been described in medical literature. These cysts result from aberrant embryological development and are usually located above the diaphragm. Their presence in relation to abdominal organs has been described in around 100 reports. We present the first report of a ciliated foregut cyst in the triangle of Calot, in relation to the common hepatic duct. The diagnostic and therapeutic implications and the malignant potential of these lesions are further discussed.

## Case presentation

**Fig. 1:**
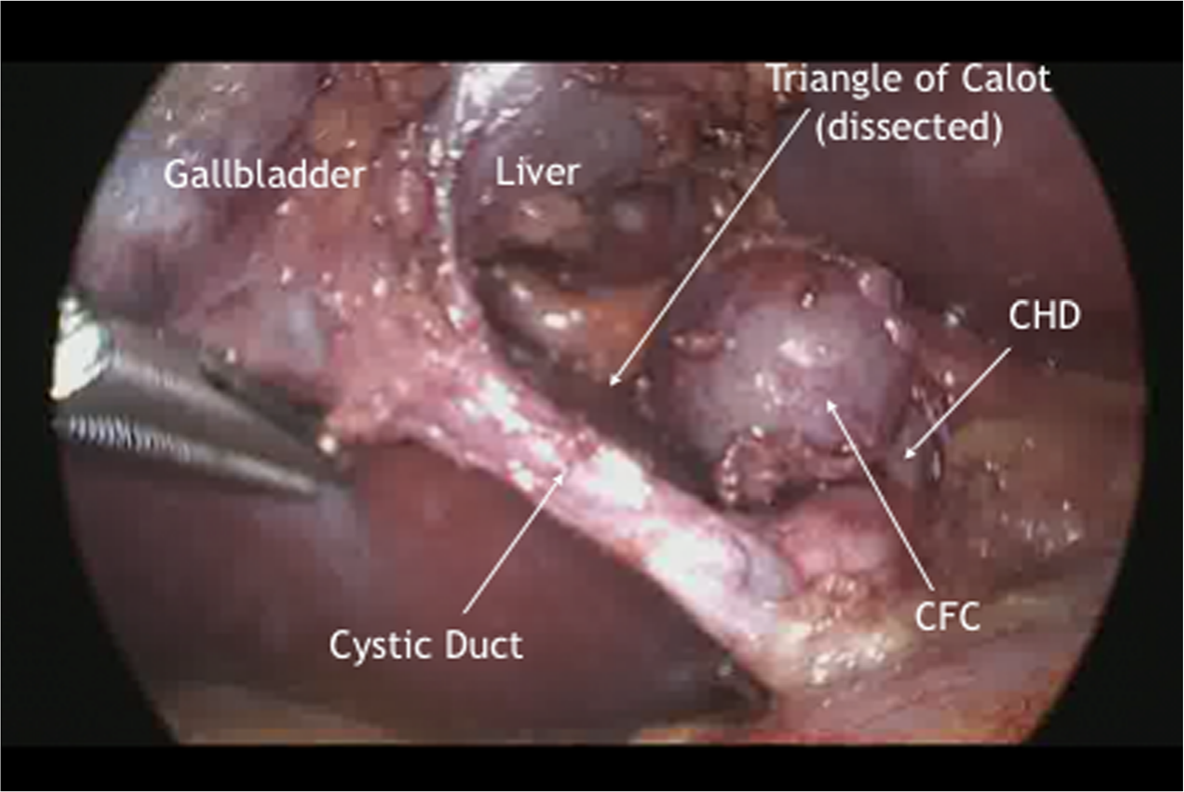
An intra-operative image showing the ciliated foregut cyst (*CFC*) within the triangle of Calot attached to the distal part of the common hepatic duct (*CHD*)

**Fig. 2:**
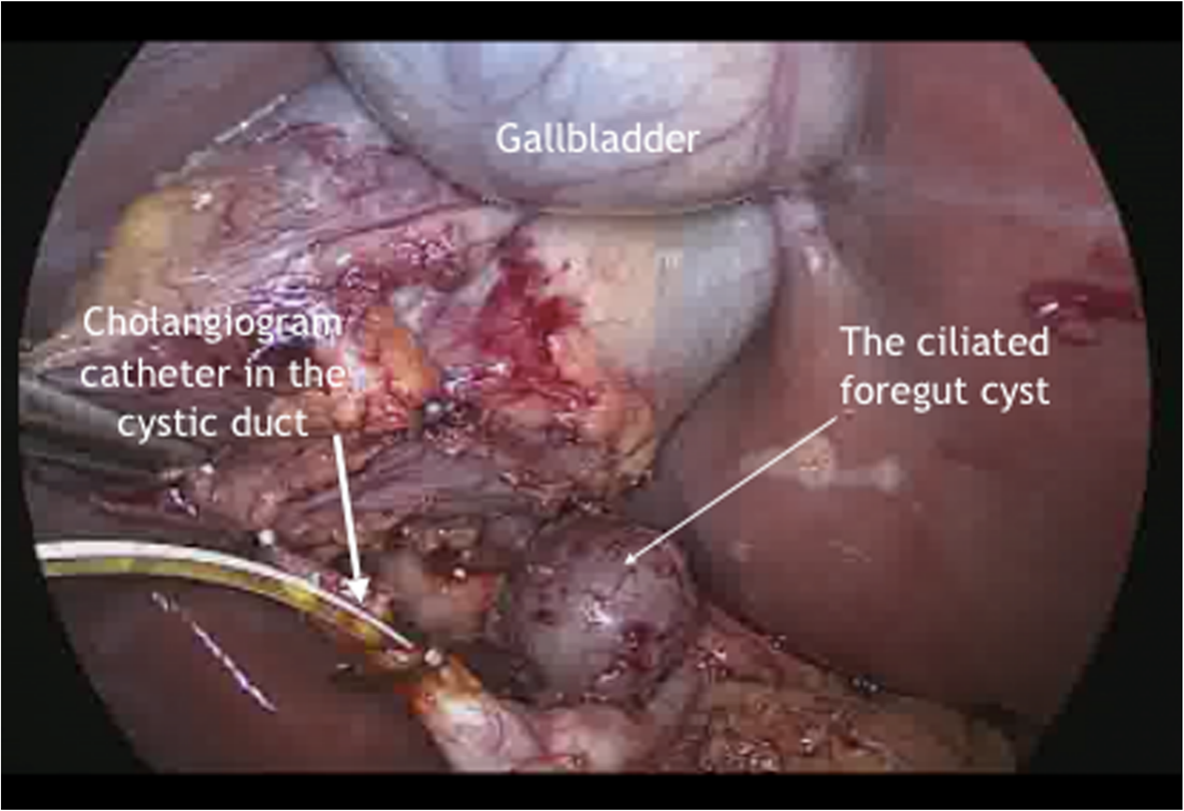
An intra-operative view of the on-table cholangiogram

**Fig. 3:**
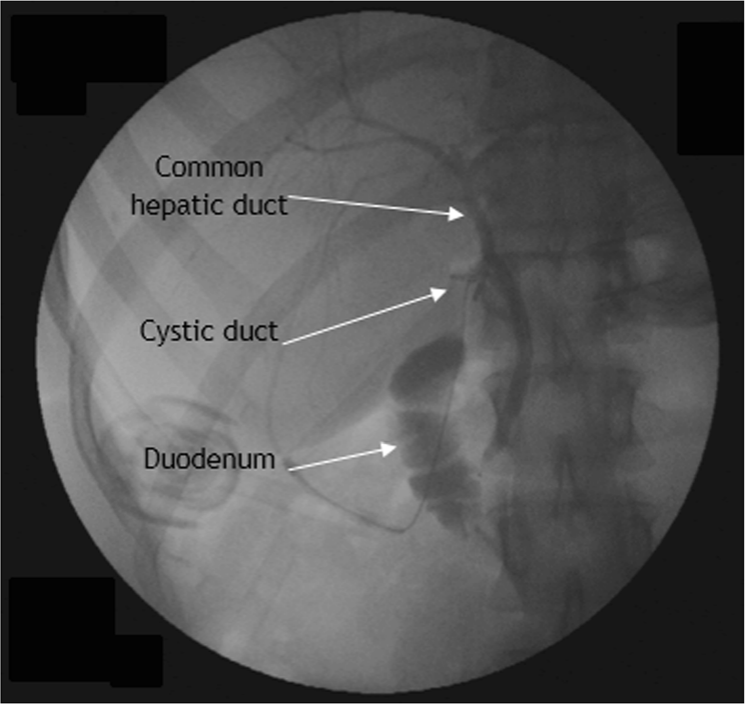
A fluoroscopic view of the on-table cholangiogram. This study shows no filling defects within the biliary tract, normal biliary anatomy and normal flow of the contrast material into the second part of the duodenum. No communication between the bile ducts and the cyst could be found

**Fig. 4:**
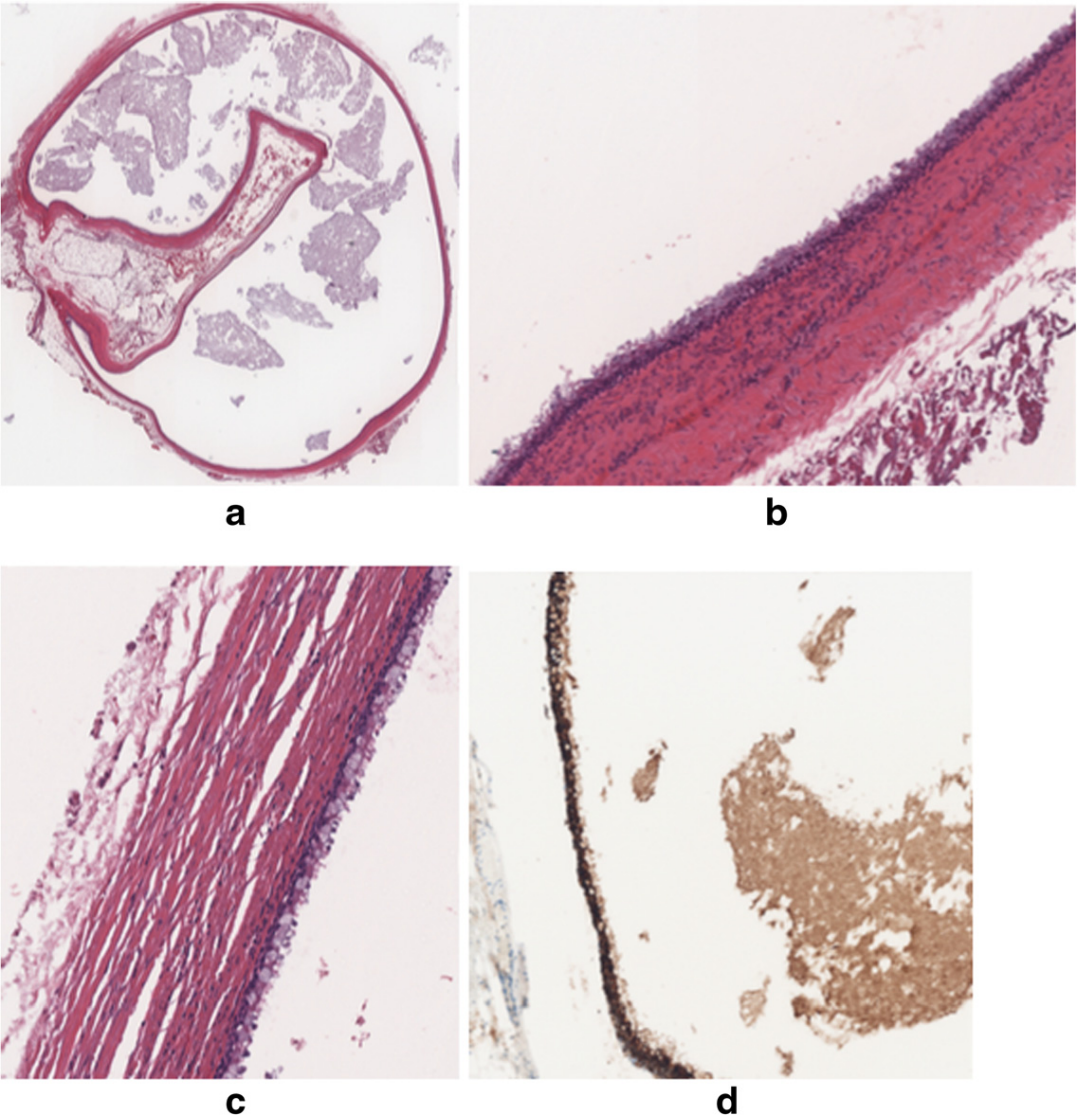
Microphotographs showing the histopathological properties of the cyst. **a** Overall view of the ciliated foregut cyst (haematoxylin and eosin, ×22 magnification). **b** Pseudostratified ciliated columnar epithelium resting on sub-epithelial connective tissue, a smooth muscle layer and an outer fibrous layer (haematoxylin and eosin, ×349 magnification). **c** Mucus-laden goblet cells in some areas of the cyst (haematoxylin and eosin, ×540 magnification).**d** CK7-positive epithelial lining (CK7 immunohistochemical stain, ×400 magnification). The epithelium is negative for CK20 and CDX2 immunohistochemical stains (not shown in the figure)

**Fig. 5:**
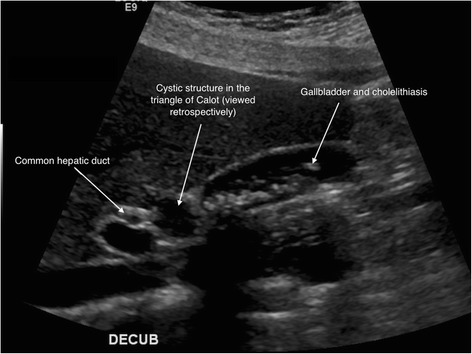
An image of the pre-operative ultrasonographic scan of the liver and biliary ducts showing multiple gallstones, normal biliary structures and portal vein and a cystic structure that could be the ciliated foregut cyst (noticed retrospectively)

A 33-year-old woman presented to the emergency department at our hospital with recurrent episodes of right-upper-quadrant pain associated with multiple episodes of vomiting. She had an ultrasonographic scan of the abdomen that showed multiple mobile stones in the gallbladder with normal intra- and extrahepatic bile ducts with no other abnormality. The blood workup was normal including liver function tests. She was operated for a laparoscopic cholecystectomy on an ambulatory surgery basis. The operation was uneventful except for the finding of a 1.5-cm nodule within the triangle of Calot (Fig. 1). This well-circumscribed, spherical lesion was attached to the common hepatic duct and embedded within the triangle of Calot with no communication to the hepatic parenchyma or gallbladder. Upon this abnormal finding, which we assumed to be an abnormally large cystic lymph node, an on-table cholangiogram (Fig. 2) was performed that showed normal biliary anatomy, no filling defects within the biliary tract and normal flow of the contrast material into the second part of the duodenum (Fig. 3). The node was bluntly and sharply dissected off the common hepatic duct, and it showed not to be related to the cystic artery and was separate from the cystic lymph node. No communication between the cystic structure and the common hepatic duct, the cystic duct or any vascular structures could be demonstrated, except for thick fibrous tissue attaching the mass to the distal part of the common hepatic duct. The operation was otherwise uneventful for any complications, and the patient was discharged on the same day. Histopathological examination showed the gallbladder to be chronically inflamed and the node to be 1.5 cm in greatest diameter. This node is a unilocular cyst (Fig. 4) lined with pseudostratified ciliated epithelium admixed with mucinous cells. Underlying this epithelial layer, connective tissue stroma, a thin layer of smooth muscle cells and an outer fibrous layer were identified. No communication or ductal structures could be found. Furthermore, immunohistochemical staining showed the cyst to be cytokeratin 7 (CK7) positive and cytokeratin 20 (CK20) and CDX2 negative. These histological findings are consistent with a ciliated foregut cyst. With this final diagnosis, the pre-operative ultrasonographic scans were reviewed retrospectively (Fig. 5). The possibility of the presence of a cystic structure separate from the gallbladder and the common hepatic duct was entertained. Obviously, such a subtle finding would have been easily missed pre-operatively taking into consideration the patient’s presentation, described above.

### Discussion

Ciliated foregut cysts (CFC) are extremely rare, benign, congenital cystic lesions that arise from the embryonic primitive foregut [1]. These cysts are most often solitary and unilocular characterised by an internal pseudostratified, ciliated, mucin-secreting, columnar epithelial lining [1]. These lesions are usually located above the diaphragm [2, 3] with a few reports of ciliated cysts in relation to abdominal organs.

During embryonic development, the differentiation of the primitive foregut cells gives rise to the oropharynx, oesophagus, larynx, tracheobronchial tree, lungs, stomach, proximal duodenum, pancreas, liver and biliary system [3–5]. The presence of cysts with a respiratory-type epithelial lining in relation to abdominal organs is aberrant. However, it is postulated that the same mechanisms underlie the development of CFC in relation to abdominal organs and the respiratory system [3, 10].

Consequently, the association of CFC with the biliary system is quite exceptional. The first description of these lesions in the gallbladder was by Kakitsubata et al. in [6]. A ciliated cyst of the common bile duct was reported by Baranger et al. in [7]. In 2000, Nam et al. were the first to introduce the term ‘ciliated foregut cyst of the gallbladder’ in their report [5]. Five other reports described ciliated cysts found within the wall of the gallbladder [2–4, 8, 9].

The differential diagnosis of hepatic CFC would include biliary cyst, parasitic cyst, mucinous cystic neoplasm and various cystic metastases, such as cystic neuroendocrine tumour or necrotic metastases. When identified in the gallbladder fossa, a pancreatic pseudocyst, choledochal cyst or gallbladder duplication should also be considered [10].

To our knowledge, this is the first description of a ciliated foregut cyst in the triangle of Calot presenting as a unilocular cyst that is attached by fibrous tissue to the common hepatic duct and is totally extramural and without any communication to the gallbladder or the hepatic ducts [10].

This case highlights the diagnostic challenges and management options posed by this pathological rarity. In this case, the pre-operative ultrasound scan could not specifically identify the presence of the cyst, and an intra-operative cholangiogram could not prove a communication between the cyst and the biliary tracts. Furthermore, there were reports on the transformation of hepatic ciliated foregut cysts into primary squamous cell carcinoma [9, 10]. The exceptional presentation of such a lesion precludes firm conclusions in this regard. As malignant potential cannot be totally excluded and in the absence of well-defined surveillance criteria, the excision of these tumours, when diagnosed, would be a rational approach mainly in young and symptomatic patients.

## Conclusions

Ciliated foregut cysts in relation to abdominal organs are a pathological rarity. This is the first report of a foregut cyst in the triangle of Calot. Due to this incidental presentation, no firm conclusions can be drawn with regard to the diagnostic workup and management plan. However, sharing histological similarities with the more common hepatic ciliated foregut cysts, which are known to have neoplastic potential, the excision of these tumours would be a rational approach mainly in young and symptomatic patients.

## Consent

Written informed consent was obtained from the patient for publication of this case report and any accompanying images. A copy of the written consent is available for review by the Editor-in-chief of this journal.
